# Functional and structural connectome-based predictive modelling of balance in elderly adults

**DOI:** 10.1038/s41598-026-43724-0

**Published:** 2026-03-17

**Authors:** Xinyu Liu, Selin Scherrer, Sven Egger, Benedikt Lauber, Wolfgang Taube, Lijing Xin

**Affiliations:** 1 Laboratory for Functional and Metabolic Imaging (LIFMET), Ecole Polytechnique Fédérale de Lausanne, EPFL AVP CP CIBM Station 6, 1015 Lausanne, Switzerland; 2 CIBM Center for Biomedical Imaging, Lausanne, Switzerland; 3 MR Imaging and Technology, Ecole Polytechnique Fédérale de Lausanne (EPFL), Lausanne, Switzerland; 4 Department of Neurosciences and Movement Science, University of Fribourg, Fribourg, Switzerland; 5 Institute of Physics, École Polytechnique Fédérale de Lausanne, Lausanne, Switzerland

**Keywords:** Motor control, Neural circuits

## Abstract

Balance control is fundamental to the quality of life among older adults, yet its neural underpinnings remain only partially understood. Despite advances in neuroimaging techniques, the neural correlates of balance are often examined at a regional level and typically restricted to either the functional or structural connectome alone. In this study, we employed connectome-based predictive modelling (CPM) for a large-scale discovery of brain connections predictive of individual balance abilities using both structural and functional connectomes in a cohort of 54 older adults. The test–retest reliability and specificity of the constructed models was evaluated using repeated-measurement data and strength performance data. Our results show that both structural and functional connectomes can successfully predict balance performance on an unstable device measured using mean sway area. A comprehensive system, encompassing motor-subcortical connections, medial-frontal and fronto-parietal networks emerged from both connectome types as consistent predictors of balance. Notably, connections with visual networks uniquely contributed to prediction in the structural but not in the functional connectomes. Structural connectomes also showed better prediction performance and test–retest reliability compared to functional connectomes. The specificity of constructed models was validated using strength performance data. In summary, our study shows that structural and functional connectomes are strong predictors of motor control abilities in challenging conditions in the elderly highlighting their interdependency and complementary roles in balance control.

**Supplementary Information:**

The online version contains supplementary material available at 10.1038/s41598-026-43724-0.

## Introduction

Maintaining balance is a motor skill essential for daily activities and mobility. It is well-recognized that balance control is achieved through a complex interplay of brain regions to integrate sensory information and generate adaptive motor output in response to changes in body orientation, thereby maintaining the center of gravity within the base of support^1^. Elderly adults are at a higher risk of impaired balance control which significantly increases their likelihood of falling. This, in turn, can lead to reduced mobility, loss of independence, and diminished quality of life^2^. Multiple factors contribute to the age-related decline in postural control and increased risk of falls, such as deteriorating vision^3^, vestibular function^4^, somatosensation^5^, and muscle function^6^. Crucially, this decline is also driven by degenerative changes in the central nervous system, such as reduced intracortical inhibition^7–9^ and alterations in brain structure and function^10^. Identifying and understanding the neural basis of balance is therefore crucial for ensuring the well-being of the aging population and developing targeted interventions and rehabilitation strategies^11^.

Recent advances in structural and functional magnetic resonance imaging (MRI) have greatly expanded our knowledge of the neural correlates of balance control. Studies using structural MRI have demonstrated that the cerebellum, basal ganglia, thalamus, hippocampus, inferior parietal cortex and frontal lobe regions are key regions associated with balance abilities (see review^12^), where training- and/or pathological-induced volumetric changes in grey or white matter were shown to correlate with changes in balance performance^13,14^. In addition, improvements in balance after training intervention have been shown to be accompanied by an upregulation of functional connectivity in the cortical motor network^15,16^, the striatum^17^, and visuospatial and executive control networks^18^. In contrast, impaired balance in various neurological disorders has been linked to decreased functional connectivity in cortical vestibular^19^, cerebellar-cortical^20^ and sensorimotor networks^21^. These findings underscore that balance control and adaptation of balancing skills relies on the dynamic structural and functional coordination among distributed brain regions. Consequently, characterizing these networks is essential for advancing our understanding of the neural basis of balance adaptation and deficits.

Despite growing evidence that balance control is governed by distributed neural networks rather than isolated brain regions, previous studies are often focused on a (hypothesis-driven) set of pre-defined regions or networks of interest, lacking a comprehensive analysis of large-scale network interactions. Connectome-based predictive modeling (CPM) has emerged as a data-driven approach for uncovering brain-behavior relationships by leveraging whole-brain connectome data^22^. CPM constructs predictive models based on whole-brain connectivity, allowing for the identification of brain connections that significantly contribute to behavioral outcomes through rigorous cross-validation procedures. This method has been successfully applied in investigations of multiple areas of the brain-behavior relationship, such as cognition^23,24^, symptoms of neurological disorders^25–27^ and intervention-induced neural adaptations ^28^. To date, however, there has been limited research using CPM specifically to investigate balance control in healthy population.

Though CPM was initially designed and predominantly applied to functional connectomes, recent studies have demonstrated its feasibility for use with both, structural and functional connectome in predicting cognitive functions, highlighting their complementary contributions^23^. It is increasingly recognized that the coupling between brain structural and functional connectome may be crucial for a better understanding of the brain-behavior relationships^29,30^. This coupling can manifest in both convergent and divergent patterns. Convergency refers to the alignment between structural and functional connections, where strong anatomical links are mirrored by coherent functional interactions. On the other hand, divergency refers to instances where structural and functional connectomes show distinct patterns. In such cases, functional connectivity may emerge from indirect interactions not directly constrained by anatomical pathways^31^. Though the coupling between structural and functional connectomes have been studied extensively through correlation analysis, it remains unclear whether combining both modalities enhances behavioral prediction by capturing unique, non-overlapping neural information (“complementarity”) or their predictive power largely overlaps (“congruency”). Thus, the application of CPM to structural as well as functional connectome in the context of balance prediction may offer valuable insights into their shared and unique neural substrates underlying postural control.

In this study, we employed multimodal neuroimaging to construct both structural and functional connectomes in a cohort of older adults, applying connectome-based predictive modeling (CPM) to predict individual balance performance on an unstable device. Our primary aim was to identify network connections predictive of individual balance performance. Furthermore, the predictive models were evaluated for test–retest reliability using longitudinal repeated measurement data and specificity using performance data from another motor modality. We hypothesize that structural and functional connectome-based prediction would capture individual differences in balance performance and that distributed connections across the whole brain contribute to the prediction. Furthermore, we explored whether combining structural and functional connectome could enhance the prediction by capturing unique neural information.

## Methods

### Study design and participants

Sixty participants aged 64–82 (71.1 ± 4.8 years old, 29 males, 31 females) were recruited in this study. All participants were cognitively unimpaired and free of any known neurological and orthopedic diseases or injuries as well as contradictions to MRI measurements. A multi-modal MRI scanning session was performed, including diffusion tensor imaging (DTI), resting-state functional MRI and structural MRI measurements. Their balance performance was assessed using a wobble board measurement (details below). All participants provided written informed consent prior to the experiments and the experiments were performed with the approval of the local ethics committee (Commission cantonale d’éthique de la recherche sur l’être humain (CER-VD); ID: ID 2021-00378). All experiments were performed in accordance with relevant guidelines and regulations. Six participants failed to complete either the balance performance measurement or the MRI measurement and were thus excluded from the analysis, resulting in 54 participants in the final analysis.

Forty-eight participants of the 54 were scanned about three months after the first scan using the same MRI protocols, which is a part of our previous longitudinal study investigating motor-training induced neural plasticity^16^. They also underwent a second measurement of balance performance. The remaining six participants failed to participate in the second scan or balance measurement. In this study, the time point 2 (the second measurement) data were used to validate the predictive models constructed using the time point 1 (the first measurement) data.

### Balance performance measurement

Balance performance was evaluated using wobble boards, on which participants stood with both feet and hands held akimbo. The devices have a rounded bottom, creating an unstable surface. An illustration of this device can be found in^32^. Participants were instructed to remain as stable as possible for twenty seconds while visually fixing a cross taped to the wall while two successful trails were recorded. Trials counted as successful if the participants did not use any external assistance and remained standing on the board. The pause between trials was approximately 1 min. The wobble boards were placed on a force plate (508 × 464 mm; OR 6–7 force platform; Advanced Mechanical Technology Inc., Watertown, MA, USA) to record the center of pressure movements. The force plate signals were sampled at a frequency of 4 kHz and amplified 4000 times (GEN 5, Advanced Mechanical Technology Inc., Watertown, MA, USA). Recordings were analyzed offline with Matlab (R2021b, The MathWorks, Inc., Natick, MA, USA). Each measurement consists of two attempts and the mean sway area (area of smallest ellipse that covers 95% of center of pressure data points) of both attempts are recorded. The lower mean sway areas in cm^2^ of the two attempts was taken as the balance performance parameter (smaller sway areas indicate better balance performance). The sway area has been used in previous studies to assess postural stability^33^ and is calculated using the following formula:

$$Area = \pi \times 2.4478 \times \sqrt {eigen\,values \,of \,the \,covariance \,matrix \,of \,COP}$$

This formula gives the 95% confidence ellipse area, which covers 95% of the center of pressure (COP) data points. The ellipse represents the spatial distribution of the sway providing a measure of the extent of postural sway.

### MRI Data acquisition

All MR measurements were performed on a 7-T/68-cm MR scanner (Magnetom, Siemens Medical Solutions, Erlangen, Germany) equipped with 80 mT/m gradients and with a single-channel quadrature transmitter and a 32-channel receiver coil (Nova Medical Inc., MA, USA).

A high-resolution MP2RAGE^34^ sequence was used to acquire structural brain image (voxel size = 0.6 × 0.6 × 0.6 mm^3^, TR/TE = 6000/4.94 ms, TI1/TI2 = 800/2700 ms, slice thickness = 0.6 mm, field of view = 192 × 192 × 154 mm^3^, acquisition time = 10 min 8 s). Resting-state functional MRI images were acquired using a 2D multi-slice gradient-echo echoplanar imaging (EPI) sequence (TR = 1550 ms, TE = 26 ms, field of view = 198 × 198 mm^2^, in-plane resolution = 1.3 × 1.3 mm^2^, slice thickness = 1.3 mm, 100 slices, GRAPPA factor = 3, acquisition time = 6 min 52 s)^35^. Diffusion-weighted MRI images were acquired using a 2D multi-slice pulsed-gradient spin-echo (PGSE) EPI sequence (TR = 5000 ms, TE = 59.8 ms, b-values = 0 (4 repetitions), 200 (6 directions), 1000 (30 directions), 2000 (60 directions) s/mm^2^, voxel size = 2.0 × 2.0 × 2.0 mm^3^, field of view = 230 × 230 × 120 mm^3^, multi-band acceleration factor = 2, GRAPPA factor = 3, partial Fourier = 6/8, acquisition time = 8 min 45 s).

### MRI data processing

#### Functional MRI data processing

Resting-state fMRI data were processed using a custom pipeline. First, the initial 5 time points of functional data were removed to ensure magnetic field stabilization. Functional and structural brain images were skull-stripped using SynthStrip^36^. The skull-stripped functional images were then corrected for head motion using MCFLIRT^37^ implemented in FSL (https://fsl.fmrib.ox.ac.uk/fsl/fslwiki/FSL). Next, susceptibility distortion correction was performed using SynBOLD-Disco^38^. In this pipeline, a “synthetic, undistorted” BOLD image that matches the geometry of structural T1w images and the T2* contrast was generated using a pre-trained neural network. This image was then used as reference for FSL TOPUP^39^ for distortion correction. The distortion-corrected images were then transformed to Montreal Neurological Institute (MNI)-152 2-mm space by first registering functional image to structural image with epi_reg functionality in FSL, followed by registering from structural space to MNI space using antsRegistrationSynQuick functionality implemented in Advanced Normalized Tools (ANTS) (https://github.com/ANTsX/ANTs). The transformed images were smoothed using a Gaussian kernel of full width at half-maximum (FWHM) of 4 mm. Next, the images in standard space were further processed with ICA-AROMA^40^, an independent component analysis (ICA) based method for removing motion-related artifacts. After this step, white matter and cerebrospinal fluid signals were regressed from the images. Finally, a high-pass filter of 0.01 Hz was applied to remove low-frequency drifts.

#### Diffusion MRI data processing

Diffusion imaging data was processed using a custom pipeline. First, skull-stripping was performed using SynthStrip. The skull-stripped images were then denoised using MP-PCA^41^ and corrected for Gibbs ringing artifact^42^. Following this step, the diffusion images were corrected for susceptibility distortion using Synb0-Disco method^43^, which uses deep learning to generate an undistorted b0 volume using a participant’s T1-weighted anatomical image. The undistorted volume was then passed to FSL’s TOPUP program to correct distortions in the acquired DWI images. After TOPUP, the images were further corrected for eddy currents using FSL’s eddy^44^.

Following pre-processing, probabilistic streamline tractography was performed using MRtrix3^45^. First, from the preprocessed DWI volumes, single-fiber response functions for white matter (WM), gray matter (GM), and cerebrospinal fluid (CSF) were estimated via dwi2response with the “dhollander” algorithm^46^. Multi-shell, multi-tissue constrained spherical deconvolution was then performed to generate fiber orientation distributions (FODs) using dwi2fod^47^. The FOD maps were corrected for bias fields and intensity inhomogeneities via mtnormalise^48^. Probabilistic tractography was performed to construct a “tractogram” via tckgen with the “iFOD2” algorithm (10 million streamlines, maximum tract length = 250 mm, FA cutoff = 0.06). The initial tractogram generated was then filtered using Spherical-deconvolution Informed Filtering of Tractograms (SIFT2) methodology. SIFT2 ensures that the (weighted) streamline reconstruction is an adequate fit of the diffusion signal at each voxel, which is itself proportional to the volume of tissue aligned in each orientation, thus providing more biologically meaningful estimates of structural connection density^49^.

#### Connectome construction

After pre-processing, whole-brain functional connectivity (FC) and structural connectivity (SC) matrices (connectomes) were constructed using the Shen 268-node atlas^50^, which divides the brain into 268 nodes organized into 10 brain networks.

Functional connectomes were constructed using the GRaph thEoreTical Network Analysis (GRETNA) toolbox^51^. Mean time series from each node in the processed functional MRI images were extracted by averaging the time series of all voxels in the node. Pairwise Pearson correlations were then computed between each pair of nodes, resulting in a 268 × 268 connectivity matrix. The elements in the connectivity matrices are Fisher’s z-transformed values of the correlation coefficients (r values).

Structural connectomes were constructed from the processed DTI data using the tck2connectome command in MRtrix3^45^. To account for differences in node sizes, streamline counts were normalized by node volume^23^.

### Connectome-based predictive modelling

We applied CPM to both, structural and functional connectomes at time point 1 for balance performance prediction following the standard pipeline^22^. Then, the constructed model is used for the data obtained at time point 2 to evaluate test–retest reliability of the CPM. The CPM approach identifies brain networks significantly contributing to behavior by using a leave-one-out cross validation approach.

#### Leave-one-out cross validation (LOOCV)

In LOOCV, we remove one participant (testing set) from the dataset and use the remaining N – 1 participants (training set) to build the predictive model. This step is repeated in an iterative manner with a different subject left out in each iteration, resulting in N predicted performance scores. The prediction effectiveness is then evaluated by the correlation between predicted scores and actual scores. We now describe the model-building process in each training step.

#### Feature selection

For each edge in the connectomes of the training set, Spearman’s rank correlation between connection strength and balance performance were calculated, resulting in a 268 × 268 matrix of correlation strength (or *p* value) between individual edges and performance. Age and sex were included as covariates. The matrix is then thresholded based on p value, where the optimal threshold is found using the threshold selection process described in^23^. Briefly, the threshold ranges from 0.0001 to 0.05 is searched, where the model stability is evaluated by prediction effect size (correlation between predicted scores and actual scores) variance within each 0.0001 interval. The optimal threshold is defined as the one that produced the highest prediction accuracy while maintaining stability. These optimal *p* value thresholds were then used as parameters for constructing predictive models.

#### Single-subject summary values

Based on connectivity matrices, we identified a positive network (edges that positively correlated with the balance metric) and a negative network (edges that negatively correlated with the balance metric). For each participant in the training set, the connectivity of all edges that survived the threshold were summed to a single value, for positive and negative networks respectively. We used sway area as the metric for balance performance where smaller sway areas indicate better balance performance. Thus, a negative correlation with the balance metric indicates a positive correlation with balance performance, and vice versa.

#### Model fitting

The edges included in each of the two predictive networks (positive or negative) were aggregated to form the final predictive features for each subject. These features were then input into corresponding linear regression models for model fitting and prediction. After the model is fitted using the training set, it is used for prediction for the left-out subject, and this process is iterated n times to obtain prediction scores for each subject. As described above, the prediction effectiveness is then evaluated by the correlation between predicted scores and actual scores.

#### Model significance test

To assess the significance level of the model, we performed a permutation test by running the leave-one-out CPM process 1000 times. We randomly shuffled the correspondence between balance performance and connectivity matrices. After each shuffle, the predicted scores and Spearman’s rho correlations between predicted and actual scores were recalculated. Finally, 1000 Spearman’s correlations comprise the null distributions of r values. *P* values were then calculated by the following formula: the number of permutated r values greater than or equal to the true r values/1000. These *p* values are then used to report the significance of models. False discovery rate correction was applied using Benjamini-Hochberg (BH) procedure across four families of statistical tests (functional-structural, positive–negative).

#### Identification of consensus connectomes

Edges present in all LOOCV iterations were considered as important for prediction and we use these edges to form a consensus connectome for reporting. To identify and compare the spatial distribution patterns of the structural and functional networks of each cognitive control subcomponent, we used BioImage Suite software (https://bioimagesuiteweb.github.io/webapp/connviewer.html#) to classify brain nodes into ten canonical networks (e.g., the medial frontal, frontoparietal, and default mode network). In this way, we were able to identify important brain networks that significantly contributed to balance performance prediction. The resulting connectomes are termed as functional/structural consensus connectomes, respectively.

To evaluate the distribution of important connections across networks, the proportion of the number of connections associated with a network in the total number of connections is calculated for each canonical network.

#### Combining functional and structural connectomes

To evaluate whether combining structural and functional connectomes could enhance the prediction performance, we constructed another model including connection strengths from both structural and functional connectomes. The model was constructed for positive and negative connectomes separately using data from time point 1. In other words, the linear regression model was modified to include positive/negative connection strengths from both functional and structural connectomes as predictors.

### Test–retest reliability

Despite the lack of an independent test set in this study, we evaluated test–retest reliability of the predictive models using the time point 2 data in our longitudinal study. Specifically, we used the models estimated from time point 1 data to predict balance performance at time point 2 using the consensus connectomes derived from time point 2 neuroimaging data as inputs. The prediction effectiveness is then evaluated in the same way (correlation between predicted scores and actual scores). This step is intended to evaluate the test–retest reliability of the model.

### Testing model specificity with strength performance

Though the approach we used could identify connectomes relevant to balance, it is still questionable whether the constructed models were predictive for balance specifically or motor abilities in general. To address this issue, we performed strength measurement of lower body parts as an additional test of model specificity.

Forty-two participants of the original 54 were included in this measurement. The torque measurements were conducted using an isokinetic machine (Cybex Norm, HUMAC, CA, USA). Participants were seated on the chair of the isokinetic device (Humac Norm, Computer Sports Medicine Inc., Stoughton, MA, USA). Their right leg was placed on the foot pedal of the machine in a supine position. To achieve the most valid power transmission possible, the participants were equipped with customized shoes directly attached to the foot pedal. Participants executed isometric ankle dorsiflexion in a neutral zero position and were verbally encouraged to maximize peak rate of torque development (RTD) by dorsiflexing as fast as possible. To familiarize themselves with the task, participants performed two familiarization trials prior to the measurement. A total of five trials were recorded with at least 15 s of rest between each. Trials failing to meet requirements (i.e. countermovement or upper body movements) were excluded from the analysis. The peak RTD was identified as the steepest point of the RTD force curve using a 20 ms moving window.

The measured RTDs were used as a metric for strength performance. After we constructed the models estimated using balance performance data, they are used to predict strength performance. If the models constructed for balance performance failed to accurately predict strength performance, the model specificity is validated.

### Robustness analysis for functional connectomes

To ensure the validity of the results derived from functional connectomes, we performed a series of robustness analyses. This includes modifying our original pipeline in three different approaches: (1) To control the effect of head motion, FSL’s fsl_motion_outliers tool was used to estimate framewise displacement (FD) values for each fMRI scan. Images with mean FD values > 0.3 mm were excluded from further analysis. In addition, mean FD was added as a covariate in addition to age and sex during feature selection; (2) repeat the analysis with unsmoothed fMRI data; (3) applying global signal regression (GSR). The remainder of the analysis pipeline was repeated consistently after each modification to assess the stability of our findings.

## Results

### Prediction using functional connectomes

**Fig. 1:**
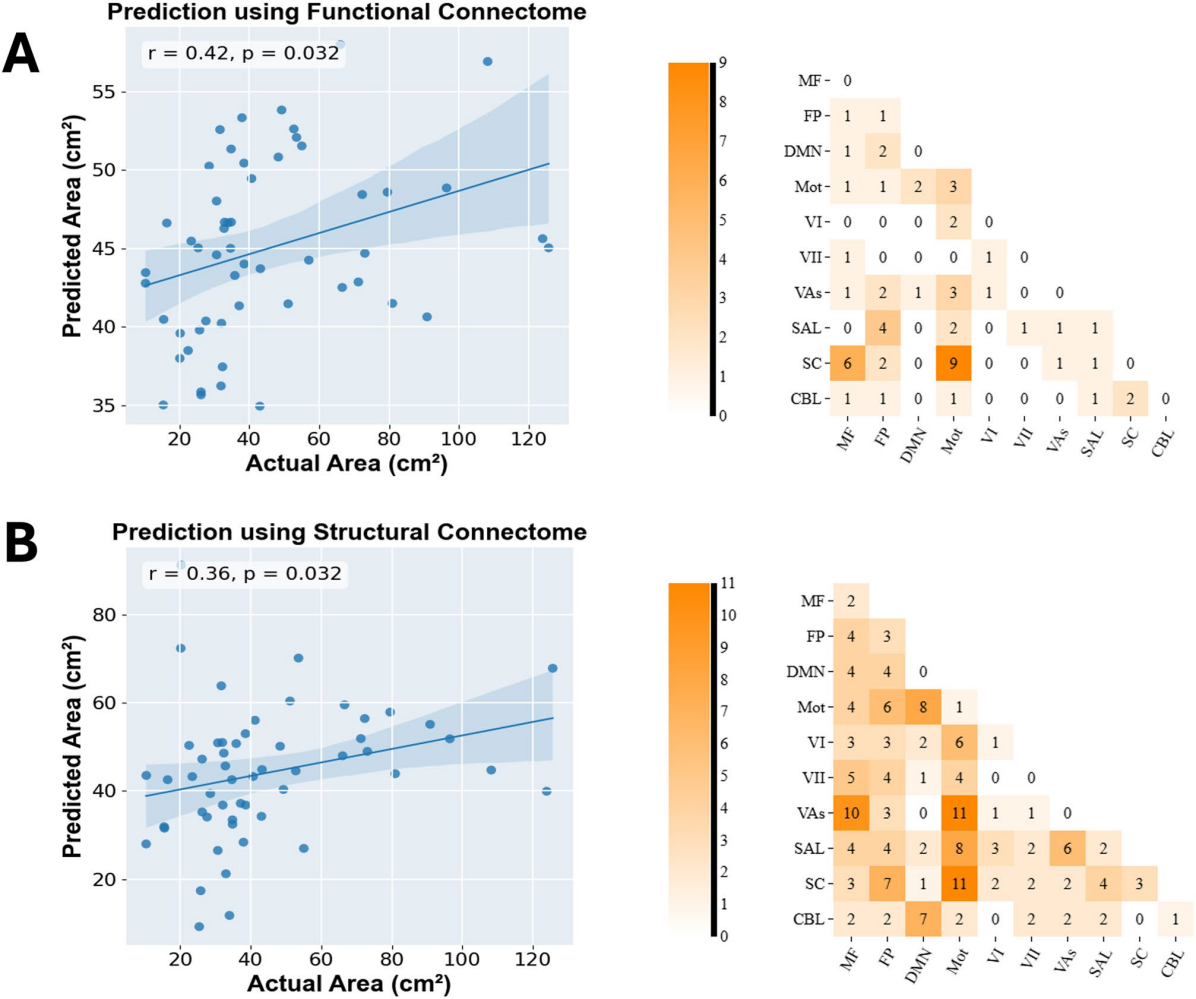
CPM prediction results at time point 1 for functional (**A**) and structural (**B**) connectomes. Top: correlations between predicted and actual sway areas, where p values here were from the permutation tests. Bottom: distribution of edges across functional networks in the consensus connectomes. MF, medial frontal network; FP, frontoparietal network; DMN, default mode network; Mot, motor network; VI, visual network 1; VII, visual network 2; Vas, visual association network; SAL, salience network; SC, subcortical; CBL, cerebellum.

For the positive functional network, the optimal p value threshold is chosen as *p* = 0.0160 (we only keep edges with p values smaller than this value). Using this value, the positive network failed to provide reliable prediction of balance performance (*p*_*permutation-corrected*_ = 0.170). For the negative functional network, the optimal *p* value threshold that is chosen as *p* = 0.0075. Using this threshold, the negative network (negatively correlated with sway area, thus positively correlated with balance performance) successfully predicted balance performance (*r* = 0.42, *p*_*permutation-corrected*_ = 0.032, Fig. 1A). Details of the model performance across different thresholds can be found in Supplementary Fig. S1).

**Fig. 2:**
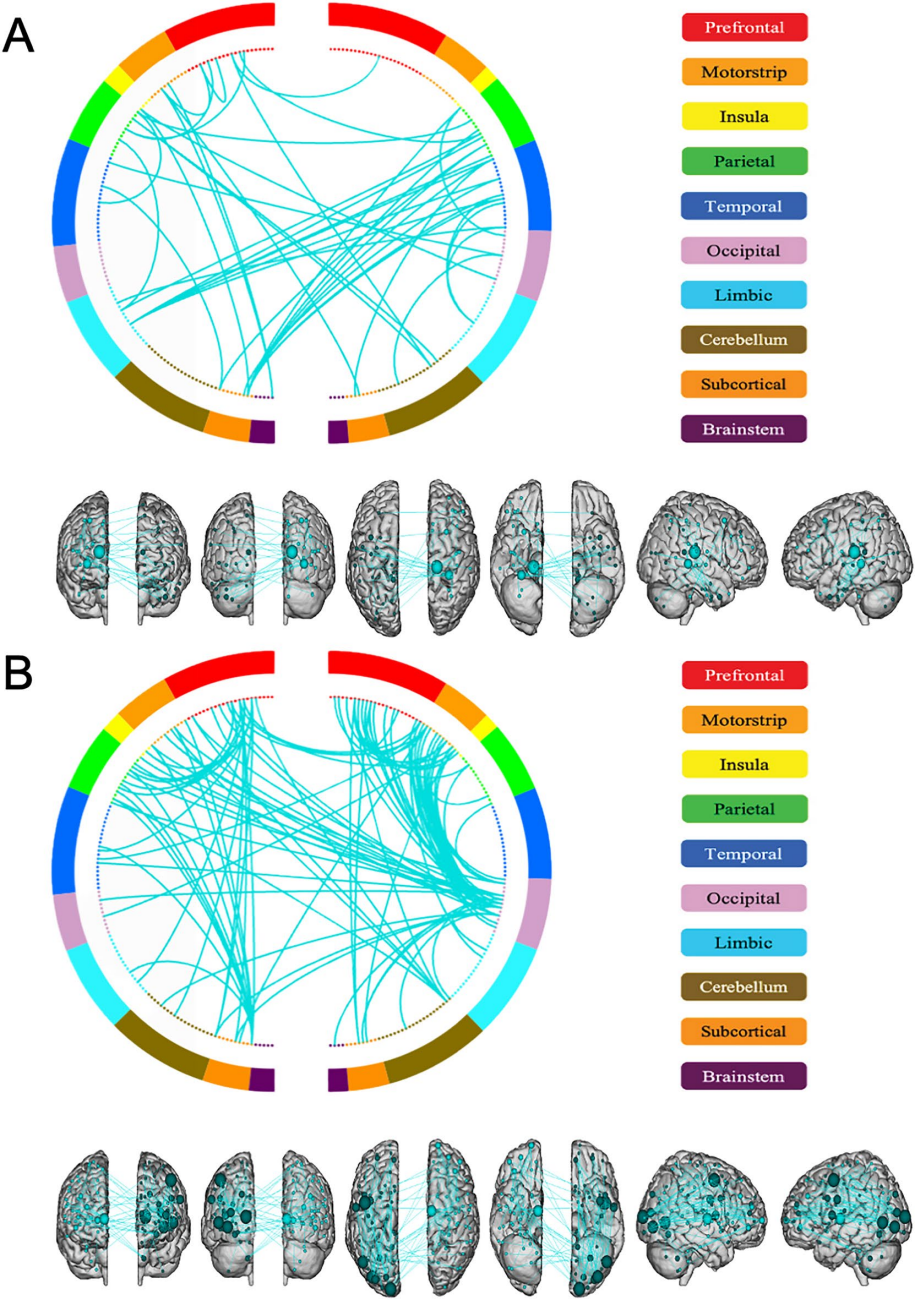
Anatomical locations of nodes and connections in the functional (**A**, displaying nodes with > 2 connections for visualization) and structural (**B**, displaying nodes with > 5 connections) consensus connectome. The image was created using the open-source tool Bioimage Suite Connectivity Viewer (https://bioimagesuiteweb.github.io/webapp/connviewer.html).

We analyzed the significant edges persistent across all negative network-based prediction iterations. In total, 58 edges were included in the functional consensus connectome. The consensus connectome was displayed from a functional and structural parcellation perspective respectively in Figs. 1A and 2A. The most implicated functional networks in balance performance predictions are motor network (22%), subcortical network (19%), fronto-parietal network (12%) and medial-frontal network (11%). The most common predictive within-network connections were found in the motor network, while the most recurrent between-network edge sets were between motor network and subcortical network, and between medial-frontal network and subcortical network. The top 20 nodes with highest degrees in the functional consensus connectome are presented in the Supplementary Table S1.

In our robustness analysis controlling FD, we found that the mean FD across all participants is 0.21 ± 0.08 mm. Two participants were removed due to excessive head motion. We found that adding FD as a covariate did not change the prediction performance (*r* = 0.48, *p*_*permutation*_ < 0.001) or consensus connectome distribution (Supplementary Fig. S2). However, using unsmoothed data and adding GSR both would result in a failure in prediction with no model can successfully predict balance performance across all searched thresholds (Supplementary Fig. S3 and Supplementary Fig. S4).

### Prediction using structural connectomes

For the positive structural network, the optimal p value threshold is chosen as *p* = 0.0250, though the constructed model failed to predict balance performance (*p*_*permutation-corrected*_ = 0.170). For the negative structural network, the optimal p value threshold is chosen as *p* = 0.0271. Using this threshold, the negative network successfully predicted balance performance (*r* = 0.36, *p*_*permutation-corrected*_ = 0.032, Fig. 1B). Details of the model performance across different thresholds can be found in Supplementary Fig. S5).

Across all iterations, 291 edges in the negative structural network consistently survived the p value threshold and are included in the structural consensus connectome. The functional and anatomical distribution of the consensus connectome were displayed in Figs. 1B and 2B, respectively. The most implicated functional systems in structural connectome-based prediction are motor network (18%), medial-frontal network (12%) and fronto-parietal network (12%). The most common predictive within-network connections were found within the fronto-parietal network and within the subcortical network, while the most frequent between-network connection sets were between motor network and subcortical network, between motor network and visual association network, and between medial-frontal network and visual association network. The top 20 nodes with highest degrees in the structural consensus connectome are presented in the Supplementary Table S2.

### Combining structural and functional connectomes

**Fig. 3:**
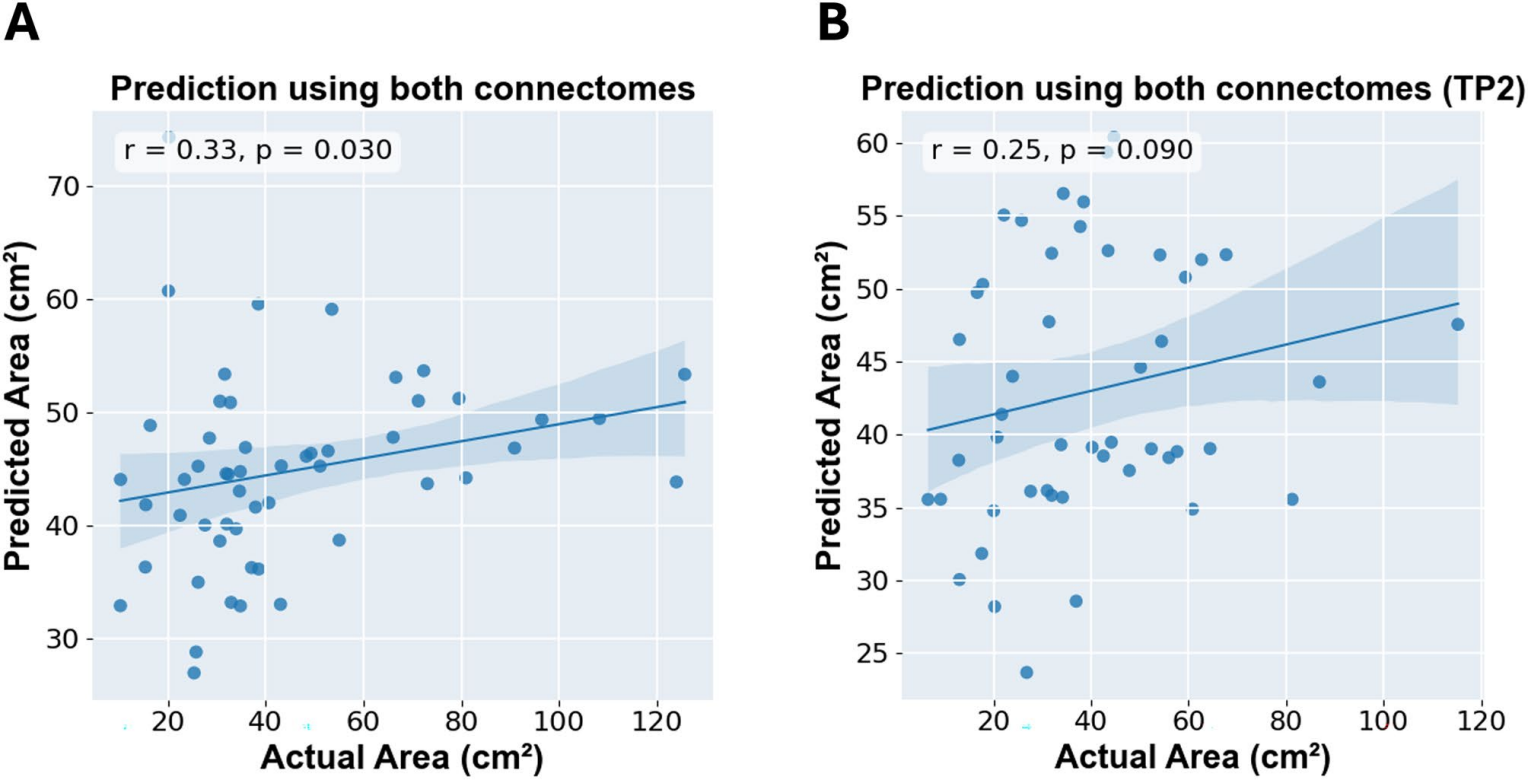
Combining both connectomes for prediction using time point 1 data (**A**) and test–retest reliability evaluation using time point 2 (TP2) data (**B**).

The combined model of negative structural and functional connectomes successfully predicted balance performance using time point 1 data (*r* = 0.32, *p*_*permutation*_ = 0.030) with a threshold of 0.262 (Fig. 3A). The threshold was chosen to be 0.263 for positive networks but failed to predict balance performance (*r* = 0.2, *p*_*permutation*_ = 0.110). Using the negative combined connectome, the test–retest reliability was evaluated, and we found a moderate test–retest performance (*r* = 0.25, *p* = 0.090) (Fig. 3B).

### Test–retest reliability

**Fig. 4:**
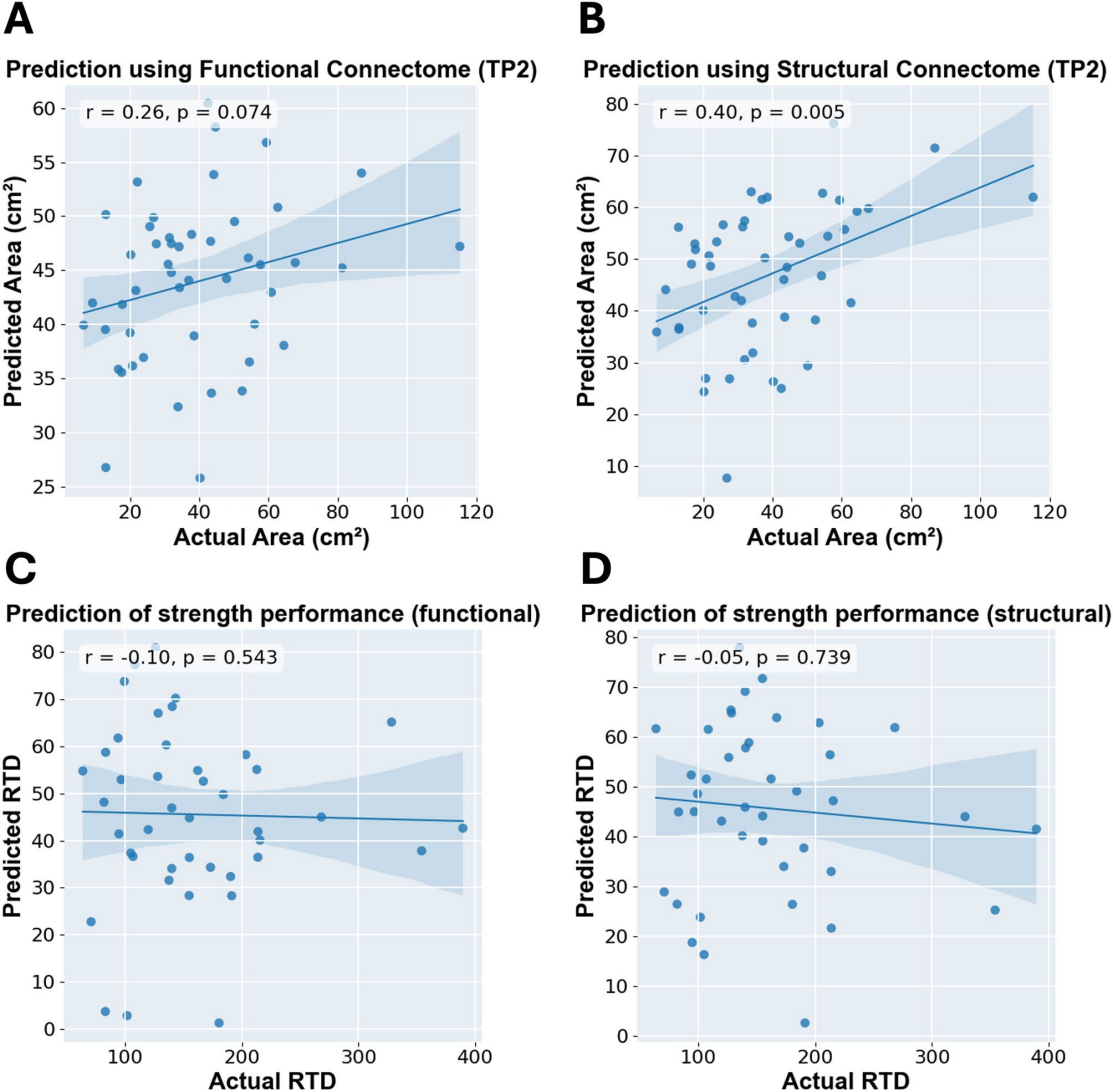
Validation experiments. (**A**) and (**B**) demonstrate prediction results of time point 2 (TP2) data using models estimated from time point 1 data for test–retest reliability. (**C**) and (**D**) demonstrate prediction results of strength performance using functional and structural consensus connectomes, respectively.

Using functional consensus connectome, the model demonstrated medium test–retest reliability when predicting time point 2 balance performance (*r* = 0.26, *p* = 0.074). Compared to functional consensus connectome, the structural consensus connectome demonstrated higher test–retest reliability by significantly predicting balance performance at time point 2 (*r* = 0.40, *p* = 0.005) (Fig. 4A and B).

### Model specificity test with strength performance

We evaluated the specificity of the model by using the model constructed from functional and structural consensus connectomes to predict strength performance. Both consensus connectomes did not successfully predict strength performance (*r* = −0.10, *p* = 0.543; *r* =  − 0.05, *p* = 0.739 for functional and structural respectively) (Fig. 4C and D).

## Discussion

In this study, we used multi-modal neuroimaging to construct functional and structural connectome-based predictive models for balance performance. We showed that both structural and functional connectome can effectively predict individual balance performance, and spatial distributions of functional and structural connections significantly linked to balance exhibited overlapping and distinct patterns. Furthermore, the effectiveness of the constructed models was successfully validated using a test–retest approach and the specificity was validated using data from another motor paradigm. Overall, these results indicate that both structural and functional connectomes can predict balance performance, and they can provide complementary information for understanding the neural underpinnings of balance when examined simultaneously.

In both structural and functional connectome-based predictions, motor-subcortical connections emerge as a prominent feature that is consistently predictive of balance performance. Specifically, increased connectivity between motor and subcortical structural and functional networks were predictive of better balance performance. This observation echoes previous functional neuroimaging and motor imagery studies where key nodes in the motor network (primary motor cortex (M1), supplementary motor area, insula etc.) and the subcortical network (thalamus and basal ganglia) were activated during motor imagery tasks where participants imagined a dynamic or reactive balance task^52,53^. Functional connectivity in motor and subcortical networks have also been reported to be upregulated following balance training interventions^16,17^. The observed relationship between motor-subcortical structural connectivity and balance also aligns with a previous study which reported reduced structural connectivity in parieto-premotor and subcortical areas in traumatic brain injury patients resulting poorer balance performance^54^. This is consistent with the notion that subcortical regions such as basal ganglia and thalamus serve as a hub that filter and modulate motor, limbic and sensory information between cortical motor networks and cerebellum^55^.

Another overlap between structural and functional consensus connectomes for prediction of balance is the rich connectivity of the fronto-parietal network and the medial-frontal network. The medial-frontal network is crucial in the monitoring and regulation of motor control^56^. For example, when an individual experiences an initial balance disturbance, the medial frontal network is responsible for utilizing sensory feedback to adjust and improve subsequent motor responses^57,58^. The fronto-parietal network, on the other hand, is recognized for its central role in top-down attentional control, response anticipation as well as motor planning and execution^59,60^. Specifically, the network is implicated in dynamic modulation of attentional resources to support balance control under challenging postural conditions^61,62^. Thus, both the medial-frontal network and the fronto-parietal network contribute to balance control by integrating sensorimotor information with attentional and executive processes to generate adaptive motor responses. Our CPM approach successfully identified these networks with complimentary roles in the regulation of balance control, suggesting that effective functional and structural connections in these networks were crucial in balance.

While posture-related neural activation patterns have been extensively studied in a localized manner using functional neuroimaging and motor imagery^63^, much less is known about the relationship between balance abilities and functional connectivity across distributed neural systems, and especially, the underlying structural brain networks. As discussed above, we found convergent patterns in both structural and functional brain networks that were consistently associated with balance performance. This overlap between significant edges from structural and functional consensus connectome reinforces the notion that functional connectivity strength is dependent on structural connections through direct fiber pathways^64^. Structure–function coupling in the brain has demonstrated gradient patterns across functional and cytoarchitectonic hierarchies^31,65^ and varies with individuals and cognitive states^66^. Furthermore, diminished structure–function coupling has been found in normal aging^67^ and in various neurological disorders^68^. Since our study identified convergent patterns in structural and functional connectomes, it would be interesting for future studies to quantitatively examine the relationship between structure–function coupling and balance.

Despite many commonalities between functional and structural consensus connectome, a notable divergence between these two is that the structural consensus connectome is featured with substantial connectivity between motor/medial frontal networks and the visual networks, which is absent in the functional consensus connectome. In other words, these connections in the structural connectome are more relevant to balance performance compared to their functional counterparts. This decoupling between structure and function aligns with the notion that despite being intrinsically constrained by structural connectivity, functional connectivity is not always dictated by structural connectivity^68,69^. A possible explanation is that structural connectivity, defined by white matter tracts, captures more static traits of the human brain, while functional connectivity fluctuates with context and task demands. For example, maintaining balance requires the integration of visual information for postural adjustment based on visual feedback^70^. During rest, these visual information pathways are not active, thus the absence of prediction power of functional connectivity may be explained by the absence of connection between the measured “baseline” functional connectivity and balance. On the other hand, the strength of structural connectivity is a more stable characteristic independent from context and task demands and thus can consistently predict balance performance. This is also evidenced by a much higher number of structural connections surviving all iterations of cross-validation and the better prediction performance of structural consensus connectome in the test–retest reliability evaluation. Thus, our findings suggest that structural and functional connectome-based CPM can provide complimentary information for the understanding of neural basis of motor behavior. Notably, combining them in the predictive model did not improve prediction performance, and resulted in worse test–retest reliability when using time point 2 data. This could suggest that the two types of connectomes are still largely congruent due to structure–function coupling, or a simple linear model could not effectively utilize the potentially unique information. Furthermore, the degraded test–retest reliability showed less stability compared to using single modality, potentially resulting from increased longitudinal variability from the two modalities. Further research could investigate alternative approaches of combining multi-modalities, such as canonical correlation analysis (CCA) based CPM and ridge regression-based CPM which have been used in integrating task connectomes for the prediction of phenotypic measures^71^.

This study has some limitations. First, a methodological constraint in this study is the moderate sample size which risks inflating model estimates. Although there are several previous successful CPM studies of similar sample sizes^24,25,28,72^, the validity of the constructed models still needs to be validated in a larger sample. We also did not collect educational level information for the participants, which could be a potential confounding factor in brain-behavior relationships. Second, an independent test set is desired for out-distribution evaluation of model effectiveness, though we evaluated test–retest reliability of our models and showed that they demonstrated medium to high reliability when tested with repeated-measurement data. Third, we only assessed one postural condition in this study (eye open on a rounded bottom platform), which may only partially reflect balance abilities. Future research is needed to fully characterize the connectivity patterns subserving balance by assessing more balance measurement paradigms and/or postural conditions. We also only measured mean sway area with two trials lasting 20 s due to limitations regarding measurement time and demands on the elderly participants. Future studies can consider using more trials and longer measurements or other markers of postural control, such as mean velocity of the center of pressure. In addition, since vestibular information relevant to postural control is primarily integrated in the right hemisphere (for right-handed participant), it would be interesting for future studies to investigate the potential differences in connectivity or integration between the two hemispheres and link them to dominance. Finally, it is important to note that the prediction performance derived from functional connectomes would diminish if using unsmoothed data or applying GSR. This is consistent with previous studies showing that both smoothing^73,74^ and GSR^75^ would have a great impact on functional brain network analysis. Future studies should therefore carefully address this issue when performing similar analyses.

In conclusion, we investigated the neural basis of balance in brain structural and functional networks using a CPM approach in an elderly cohort. Both types of networks successfully predicted balance performance, and the resulting consensus connectome depicts a comprehensive mapping of balance abilities in brain structural and functional networks, mainly encompassing motor-subcortical connections, medial-frontal and fronto-parietal networks. We further showed that structural and functional connectomes can provide complimentary information about neural substrates of behavior.

## Supplementary Information

Supplementary Information 1.

Supplementary Information 2.

## Data Availability

The data used in this study is available in supplementary information.
