# Supplementary material for: Functional and structural connectome-based predictive modelling of balance in elderly adults
Source: Sci Rep. 2026 Mar 17;16:13954. doi: 10.1038/s41598-026-43724-0 (PMC13133400; doi:10.1038/s41598-026-43724-0)
Supplement: Supplementary file 2 — Supplementary Information 2. [file 41598_2026_43724_MOESM2_ESM.docx]

**Functional and structural connectome-based predictive modelling of balance in elderly adults**

Xinyu Liu^1,2,3^, Selin Scherrer^4^, Sven Egger^4^, Benedikt Lauber^4^, Wolfgang Taube^4^, Lijing Xin*^2,3,5^


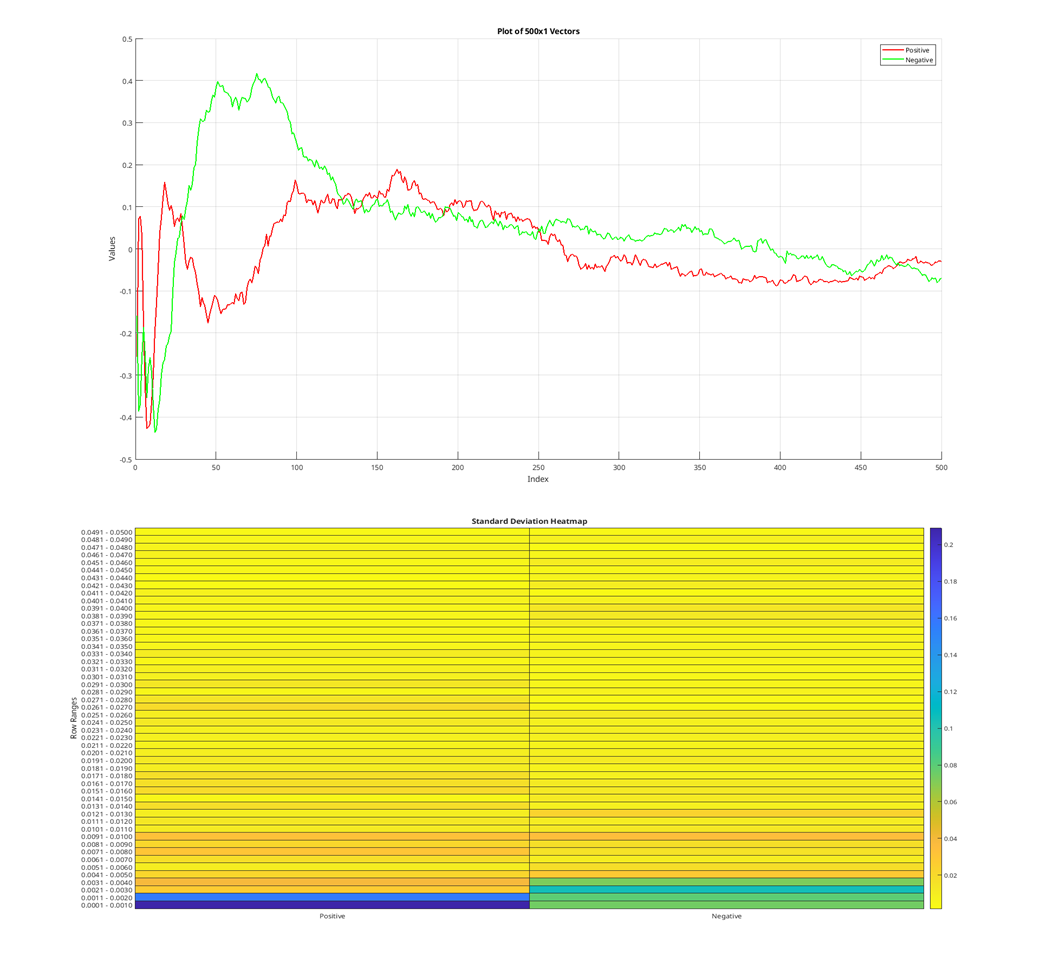


Figure S1. Performance of the model derived from functional connectome under different thresholds (top) and standard deviation across threshold range of 0.001 (bottom).


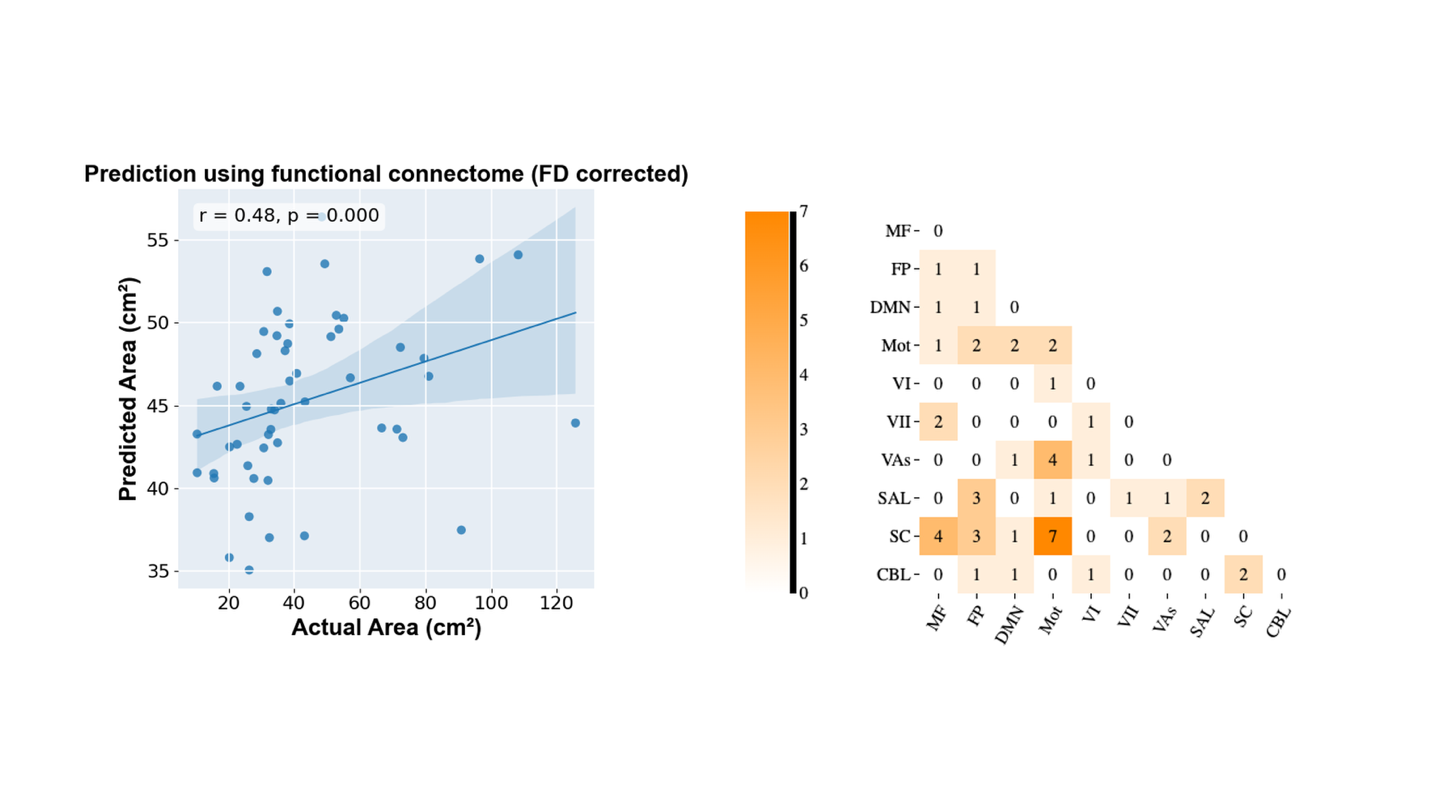


Figure S2. Prediction performance and consensus connectome derived from functional connectome with framewise displacement (FD) as an additional covariate.


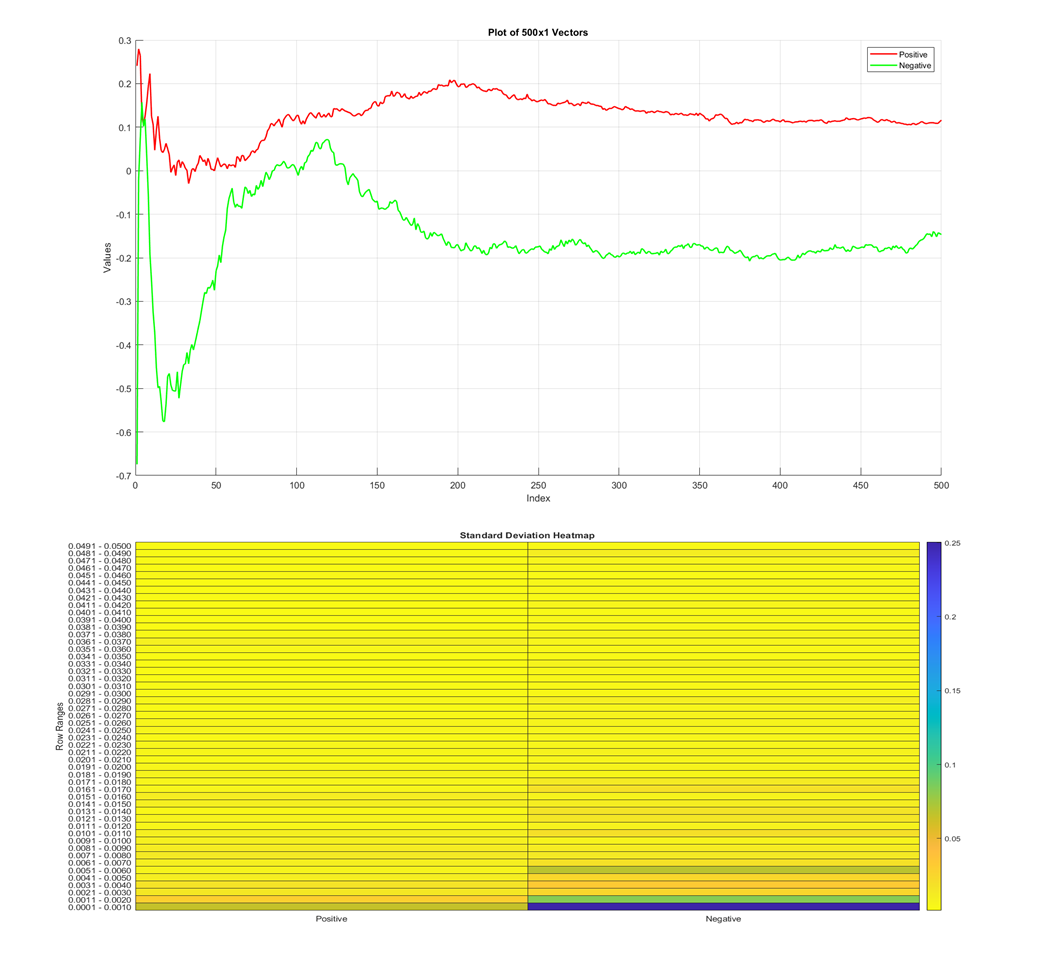


Figure S3. Performance of the model derived from functional connectome using unsmoothed data under different thresholds (top) and standard deviation across threshold range of 0.001 (bottom).


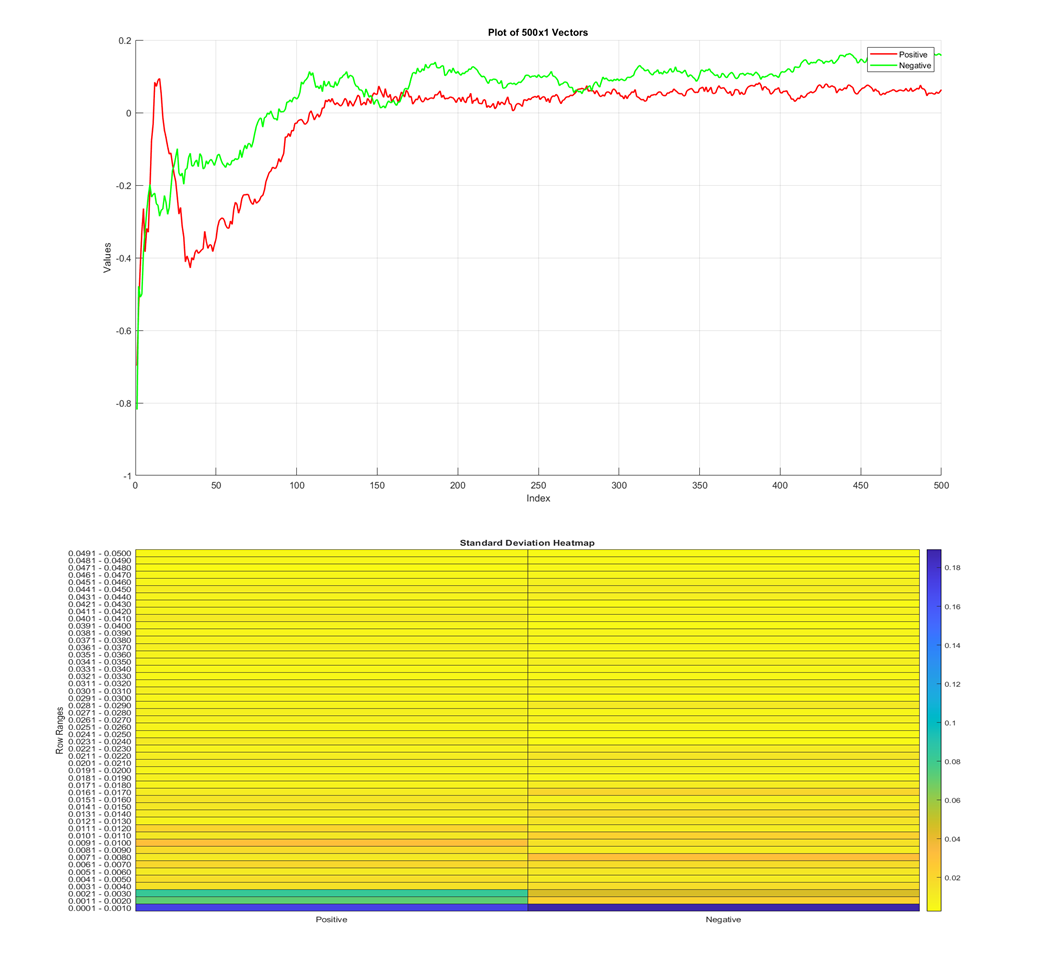


Figure S4. Performance of the model derived from functional connectome with global signal regression (GSR) under different thresholds (top) and standard deviation across threshold range of 0.001 (bottom).


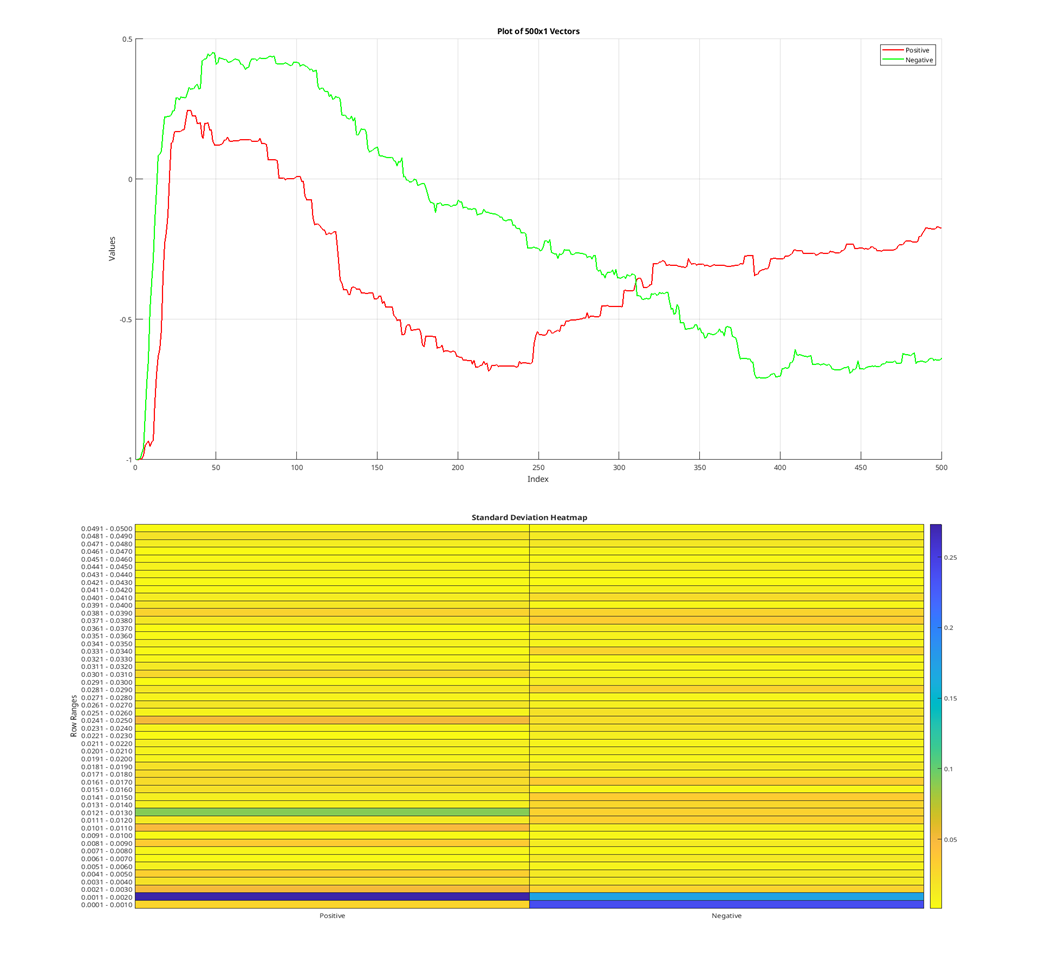


Figure S5. Performance of the model derived from structural connectome under different thresholds (top) and standard deviation across threshold range of 0.001 (bottom).

| **Number** | **Node ID** | **Details** |
| --- | --- | --- |
| 1 | 127 | Degree=7 (MNI=12.26,-27.74,13.5, Lobe=R-Subcortical) |
| 2 | 93 | Degree=5 (MNI=28.82,-36.92,-0.03, Lobe=R-Limbic) |
| 3 | 227 | Degree=3 (MNI=-7.47,-42.12,13.32, Lobe=L-Limbic) |
| 4 | 185 | Degree=3 (MNI=-38.01,6.07,-37.86, Lobe=L-Temporal) |
| 5 | 30 | Degree=3 (MNI=25.22,12.41,49.39, Lobe=R-MotorStrip) |
| 6 | 242 | Degree=2 (MNI=-30.17,-80.21,-40.35, Lobe=L-Cerebellum) |
| 7 | 209 | Degree=2 (MNI=-48.34,-67.39,1.14, Lobe=L-Occipital) |
| 8 | 202 | Degree=2 (MNI=-29.96,-5.77,-40.93, Lobe=L-Temporal) |
| 9 | 195 | Degree=2 (MNI=-37.84,-13.23,-29.26, Lobe=L-Temporal) |
| 10 | 194 | Degree=2 (MNI=-49.31,-4.7,-37.37, Lobe=L-Temporal) |
| 11 | 190 | Degree=2 (MNI=-57.62,-6.37,-22.69, Lobe=L-Temporal) |
| 12 | 181 | Degree=2 (MNI=-59.51,-25.89,21.9, Lobe=L-Parietal) |
| 13 | 178 | Degree=2 (MNI=-9.83,-66.34,55.14, Lobe=L-Parietal) |
| 14 | 170 | Degree=2 (MNI=-37.75,-12.86,-1.43, Lobe=L-Insula) |
| 15 | 133 | Degree=2 (MNI=7.47,-34.18,-37.32, Lobe=R-Brainstem) |
| 16 | 120 | Degree=2 (MNI=21.22,-36.39,22.63, Lobe=R-Subcortical) |
| 17 | 89 | Degree=2 (MNI=7.83,-23.07,44.93, Lobe=R-Limbic) |
| 18 | 43 | Degree=2 (MNI=31.62,-60.79,49.21, Lobe=R-Parietal) |
| 19 | 40 | Degree=2 (MNI=43.34,-10.81,49.58, Lobe=R-Parietal) |
| 20 | 38 | Degree=2 (MNI=32.42,-39.19,49.58, Lobe=R-Parietal) |

Table S1. The top 20 nodes with highest degrees in the functional consensus connectome.

| **Number** | **Node ID** | **Details** |
| --- | --- | --- |
| 1 | 212 | Degree=12 (MNI=-10.83,-98.14,7.63, Lobe=L-Occipital) |
| 2 | 165 | Degree=12 (MNI=-45.8,-0.43,49.28, Lobe=L-MotorStrip) |
| 3 | 210 | Degree=11 (MNI=-36.02,-84.15,-3.91, Lobe=L-Occipital) |
| 4 | 209 | Degree=11 (MNI=-48.34,-67.39,1.14, Lobe=L-Occipital) |
| 5 | 128 | Degree=10 (MNI=5.46,-9.67,5.24, Lobe=R-Subcortical) |
| 6 | 204 | Degree=8 (MNI=-31.63,-87.16,12.5, Lobe=L-Occipital) |
| 7 | 168 | Degree=8 (MNI=-39.09,1.71,9.54, Lobe=L-Insula) |
| 8 | 159 | Degree=8 (MNI=-58.09,-5.56,27.19, Lobe=L-MotorStrip) |
| 9 | 208 | Degree=6 (MNI=-16.79,-84.92,33.04, Lobe=L-Occipital) |
| 10 | 207 | Degree=6 (MNI=-25.9,-63.14,-12.25, Lobe=L-Occipital) |
| 11 | 157 | Degree=6 (MNI=-46.23,7.93,28.58, Lobe=L-Prefrontal) |
| 12 | 6 | Degree=6 (MNI=14.56,64.73,3.64, Lobe=R-Prefrontal) |
| 13 | 237 | Degree=5 (MNI=-8.7,-50.56,-39.56, Lobe=L-Cerebellum) |
| 14 | 144 | Degree=5 (MNI=-28.81,50.12,21.68, Lobe=L-Prefrontal) |
| 15 | 140 | Degree=5 (MNI=-5.96,48.09,11.72, Lobe=L-Prefrontal) |
| 16 | 11 | Degree=5 (MNI=37.62,35.39,31.09, Lobe=R-Prefrontal) |
| 17 | 9 | Degree=5 (MNI=28.88,51.14,18.68, Lobe=R-Prefrontal) |
| 18 | 8 | Degree=5 (MNI=44.56,46.19,-4.9, Lobe=R-Prefrontal) |
| 19 | 214 | Degree=4 (MNI=-22.27,-96.64,-10.06, Lobe=L-Occipital) |
| 20 | 213 | Degree=4 (MNI=-14.72,-83.97,-13.06, Lobe=L-Occipital) |

Table S2. The top 20 nodes with highest degrees in the structural consensus connectome.
